# Mercury in Commercial Fish: Optimizing Individual Choices to Reduce Risk

**DOI:** 10.1289/ehp.7315

**Published:** 2004-12-07

**Authors:** Joanna Burger, Alan H. Stern, Michael Gochfeld

**Affiliations:** ^1^Division of Life Sciences, Rutgers University, Piscataway, New Jersey, USA; ^2^Environmental and Occupational Health Sciences Institute, Consortium for Risk Evaluation with Stakeholder Participation, and School of Public Health, Rutgers University/University of Medicine & Dentistry of New Jersey-Robert Wood Johnson Medical School, Piscataway, New Jersey, USA; ^3^Division of Science, Research, and Technology, New Jersey Department of Environmental Protection, Trenton, New Jersey, USA; ^4^Environmental and Occupational Medicine, University of Medicine & Dentistry of New Jersey-Robert Wood Johnson Medical School, Piscataway, New Jersey, USA

**Keywords:** commercial fish, consumption, fish, mercury, New Jersey, risk assessment, FDA

## Abstract

Most attention to the risks from fish consumption has focused on recreational anglers and on fish caught by individuals, but the majority of fish that people eat are purchased from commercial sources. We examined mercury levels in three types of fish (tuna, flounder, bluefish) commonly available in New Jersey stores, sampling different regions of the state, in communities with high and low per capita incomes, and in both supermarkets and specialty fish markets. We were interested in species-specific levels of mercury in New Jersey fish and whether these levels were similar to data generated nationally by the Food and Drug Administration (FDA; mainly from 1990 to 1992) on the same types of fish. Such information is critical for providing public health advice. We were also interested in whether mercury levels in three common species of fish differed by region of the state, economic neighborhood, or type of store. We found significant species differences, with tuna having the highest levels and flounder the lowest levels. There were no significant differences in mercury levels as a function of type of store or economic neighborhood. There was only one regional difference: flounder from fish markets along the Jersey shore had higher mercury levels than flounder bought in other markets. We also examined mercury levels in six other commonly available fish and two shellfish from central New Jersey markets. There were significant differences in availability and in mercury levels among fish and shellfish. Both shrimp and scallops had total mercury levels < 0.02 ppm (wet weight). Large shrimp had significantly lower levels of mercury than small shrimp. For tuna, sea bass, croaker, whiting, scallops, and shrimp, the levels of mercury were higher in New Jersey samples than those reported by the FDA. Consumers selecting fish for ease of availability (present in > 50% of markets) would select flounder, snapper, bluefish, and tuna (tuna had the highest mercury value), and those selecting only for price would select whiting, porgy, croaker, and bluefish (all with average mercury levels < 0.3 ppm wet weight). Flounder was the fish with the best relationship among availability, cost, and low mercury levels. We suggest that state agencies responsible for protecting the health of their citizens should obtain information on fish availability in markets and fish preferences of diverse groups of citizens and use this information to select fish for analysis of contaminant levels, providing data on the most commonly eaten fish that will help people make informed decisions about risks from fish consumption.

Fish are an important source of protein for many people throughout the world, and their importance in the diet has increased among health-conscious Americans. Not only are fish an important source of nutrients, but fishing is a popular pastime (Burger 2002; Burger et al. 1992, 1993; Knuth et al. 2003; Toth and Brown 1997), in urban as well as in rural areas (Burger et al. 1999, 2001b; Ramos and Crain 2001). Fish provide omega-3 (n-3) fatty acids that reduce cholesterol levels and the incidence of heart disease, stroke, and preterm delivery (Anderson and Wiener 1995; Daviglus et al. 2002; Patterson 2002).

However, contaminant levels, particularly methyl mercury and polychlorinated biphenyls (PCBs), are sufficiently high in some fish to cause adverse human health effects in people consuming large quantities [Hightower and Moore 2003; Hites et al. 2004; Institute of Medicine (IOM) 1991; Stern 1993]. Fish consumption is the only significant source of methyl mercury in the public (Rice et al. 2000). Methyl mercury is reported to counteract the cardioprotective effects (Guallar et al. 2002; Rissanen et al. 2000; Salonen et al. 1995) and to damage developing fetuses and young children [National Research Council (NRC) 2000]. Maternal exposures can threaten the fetus because chemicals can be transferred to the developing fetus (Gulson et al. 1997, 1998). There is a positive relationship between mercury and PCB levels in fish, fish consumption by pregnant women, and deficits in neurobehavioral development in children (IOM 1991; Jacobson and Jacobson 1996; Lonky et al. 1996; NRC 2000; Schantz 1996; Schantz et al. 2003; Sparks and Shepherd 1994; Stern et al. 2004). There is also a decline in the fecundity of women who consume large quantities of contaminated fish from Lake Ontario (Buck et al. 2000). Mercury in fish has been featured in the media frequently, and people are faced with conflicting information about the risks and benefits of consuming fish (Consumer Reports 2001; Rauber 2001).

State agencies respond to the risk of chemicals in fish by issuing consumption advisories to inform the public about possible risks (especially to at-risk populations, such as pregnant women and children). The number of fish advisories due to chemicals, such as mercury and PCBs, has increased in the United States over the last decade [U.S. Environmental Protection Agency (EPA) 2004]. With few exceptions, state advisories do not provide information on the risk from consuming fish purchased commercially. Some states, such as New York, specifically highlight that the advisories are not for fish and game sold in markets (New York State Department of Health 2002). Recently the U.S. Food and Drug Administration (FDA 2001, 2004) issued a series of consumption advisories regarding methyl mercury that suggested that pregnant women and women of childbearing age who may become pregnant should limit their fish consumption, avoid eating four types of marine fish (shark, swordfish, king mackerel, tilefish) and limit their consumption of all other low-mercury fish to 12 ounces/week (FDA 2001). These recent FDA (2001, 2003) advisories have raised concern about the safety of fish available in supermarkets, yet there are very few data on mercury levels in commercial fish, particularly for fish expected to have low levels.

In this study we examined total mercury levels in fish in New Jersey. We used a two-tiered approach: *a*) examination of mercury levels in tuna, bluefish, and flounder purchased over a broad geographical range stratified by region, economics, and store type; and *b*) examination of mercury levels in a range of different fish and shellfish purchased in central New Jersey. We were interested in species-specific levels of mercury in New Jersey fish and whether these levels were similar to data generated nationally by the FDA on the same species (mainly from 1990 to 1992). A determination of whether national data on mercury concentrations by commercial fish species represents concentrations found in local fish can help public health providers and state health officials design their health and consumption advisories. New Jersey was specifically interested in whether the mercury levels in fish commonly sold in the state were in the range where issuing consumption advisories should be considered.

We examined different regions of New Jersey because the sources of the fish might differ. That is, fish sold in stores in southern New Jersey often comes from fish markets in Philadelphia, Pennsylvania, while fish in northern New Jersey often comes from the Fulton Fish Market in New York, New York. Thus, commercial fish enter New Jersey markets from several sources: the Fulton Fish Market, the Philadelphia fish market, commercial landings along the New Jersey coast, supermarket wholesalers, and party and charter boats. Further, fish caught locally (such as flounder and bluefish) often comes from the nearest fishing ports. Similarly, upscale and downscale markets may obtain their fish from different sources, particularly for locally available fish. Thus, it is important to understand whether mercury levels might differ in fish purchased in different regions of the state. We initially selected the three types of fish, tuna, bluefish, flounder, based on their widespread availability and the belief that they are commonly consumed and would represent high, medium, and low mercury concentrations (National Fisheries Institute 2004). Other fish were selected to represent commonly available species and those we expected would have low levels of mercury. One of our objectives was to provide data to agencies and the public on species that might pose little risk from mercury, thus providing positive information that could inform personal choices.

Fish consumers face a series of choices regarding whether to eat fish they catch or commercial fish, which species to eat, what trophic level or size of fish to eat, and how much fish to eat. To make these decisions, they must know the levels of contaminants in the fish that are commercially available. The advisories promulgated by state agencies and the FDA deal with fish that have high mercury levels and often do not provide information on fish that may be low in mercury. This study partly addresses this issue. We also combined information on availability and price with mercury levels to consider how people might reduce their risk within their local community.

## Methods

Our overall research design was to *a*) determine fish availability (and price) in the state generally (Burger et al. 2004); *b*) buy three types of fish from supermarkets and fish markets throughout the state, in towns with higher and lower socioeconomic status (SES); *c*) use the information on availability and hypothesized mercury levels to select six additional fish and two shellfish for mercury analysis to provide information on a broader range of species; *d*) determine the total mercury in these fish and shellfish; and *e*) compare the mercury data from this study with that available from the FDA that is otherwise used by state health departments and the public for guidance. The FDA generally obtains its fish by random, geographically stratified sampling (Yess 1993), combined with data gathered incidentally from inspections.

New Jersey is commonly divided into regions for administrative purposes, including the relatively urbanized north and the very rural south, as well as a large, central suburban region. For the fish availability (and price) aspect of the study (Burger et al. 2004), we visited 57 markets and fish markets in New Jersey, selected randomly from a stratified design that included four regions (north, central, south, coast), high and low SES towns, and supermarkets/fish markets. Stores were visited three times, and the fish species selected for this study were available all three times; however, a more detailed study of fish availability on a yearly basis would provide information on how availability differs seasonally, especially for winter versus summer. At a number of markets we asked about sources of fish, but the general response tied back only to the immediate suppliers. Because markets were surveyed from July through October, the data represent this time period.

For collection of fish for mercury analysis, we selected one town of higher and one of lower SES in each of the three regions and randomly selected individual stores from New Jersey’s Seafood and Fish Index Page (International Purveyor Index 2002). Both the towns within each region and the markets/ supermarkets within each town were selected randomly from those available. We defined “high” SES as above the median per-capita income for that region, and “low” SES as below the median per-capita income, and we used the U.S. Census Bureau (2000) data for per-capita income. Once we had divided the towns in New Jersey into high and low SES, we randomly selected the towns within each region for sampling. We then collected fish from two supermarkets and two fish markets in each town. Supermarkets were large chain stores selling a range of food and other grocery items, and fish markets sold primarily fish. Only fish markets were sampled along the shore, and these were mainly in shore communities with a high number of summer residents. Although we tried to balance the sample sizes from each geographical region, from high to low SES, and from fish market/supermarket, this was not always possible. In addition, we purchased the same three fish types in fish markets in the coastal area from Sandy Hook to Cape May. All purchases were made between July and October 2003.

From each market we purchased a fillet of tuna, flounder, and bluefish. Because we purchased only fillets, we do not provide data on the basis of fish size. Tuna steaks were mainly identified as yellowfin tuna (*Thunnus albacaras*), although verification to the species level is not certain. A variety of flatfish are sold under the rubric of flounder, and these may come from New Jersey waters or from remote parts of the globe. Bluefish (*Pomatomus saltatrix*) is a popular east coast sport fish and in the past decade has become widely available in stores. Tuna are large predatory fish; bluefish are medium sized predatory fish; and flounder are bottom-dwelling fish, usually reported to be low in mercury (FDA 2001). We also bought fillets of six other species of fish from markets in central New Jersey, representing widely available fish in New Jersey markets. We also purchased scallops, and large (mean mass of 20 ± 4 g) and small (mean of 8 ± 1 g) shrimp. All fish collected for this study were fresh, although we also present information on canned tuna (after Burger and Gochfeld 2004).

We analyzed mercury at the Environmental and Occupational Health Sciences Institute of Rutgers University. A 2-g (wet weight) sample of fish tissue was digested in ultrex ultrapure nitric acid in a microwave using a digestion protocol of three stages of 10 min each under 50, 100, and 150 lb per square inch (3.5, 7, and 10.6 kg/cm^2^) at 80× power. Digested samples were subsequently diluted in 100 mL deionized water. All laboratory equipment and containers were washed in 10% HNO_3_ solution before each use (Burger et al. 2001a).

Mercury was analyzed by the cold vapor technique using the Portable Zeeman Lumex (RA-915) mercury analyzer (Ohio Lumex Co., Twinsburg, OH), with an instrument detection level of 0.2 ng/g, and a matrix level of quantification of 0.002 μg/g. All concentrations are expressed in parts per million (equal to micrograms per gram) of total mercury on a wet-weight basis. In another study (Burger et al. 2001c) we found that the dry weight ranged from 23% to 33% of the corresponding wet weight (i.e., water content of 67–77%) for 11 species of fish. Many studies have shown that almost all of the mercury in fish tissue is methyl mercury, and 90% is a reasonable approximation of this proportion, which does vary somewhat among fish types and laboratories. We used a DORM-2 Certified dogfish tissue (National Research Council of Canada, Institute of Environmental Research and Technology, Ottawa, Ontario, Canada) as the initial calibration verification standard. Recoveries between 90 and 110% were accepted to validate the calibration. All specimens were run in batches that included blanks, a standard calibration curve, two spiked specimens, and one duplicate. The accepted recoveries for spikes ranged from 85 to 115%; no batches were outside of these limits. We analyzed each digested fish sample twice, with agreement of ± 5%. In addition, 10% of samples were digested twice and analyzed as blind replicates (with agreement within 15%). For further quality control, a random subset totaling 12% of samples was sent to the Quebec Laboratory of Public Health. The correlation between the two laboratories was 0.92 (*p* < 0.0001).

We used Kruskal-Wallis nonparametric one-way analysis of variance (ANOVA; generating a chi-square statistic) to examine differences among fish species and locations. We also used ANOVA with Duncan multiple range tests to identify the significant differences (SAS Institute 1995). The level for significance was designated as *p* < 0.05, but values up to *p* < 0.10 are presented to allow the reader to evaluate whether increased sample sizes would have resulted in significance.

## Results

There were significant differences in mercury levels among tuna, bluefish, and flounder, with tuna having the highest levels and flounder the lowest levels (χ^2^ = 26.3, *p* < 0.001). However, for all three species, there were few differences in mercury as a function of region, type of market, and economic neighborhood (Table 1). Indeed, there was remarkably little variation in mercury levels among fish types (i.e., low standard errors). From a risk perspective, knowing the percentage of fish that may have mercury levels > 0.3 or 0.5 ppm may be important in their selection process. For fresh tuna, the species with the highest mercury levels, 42% of the fillets had mercury levels > 0.5 ppm (Table 2).

There were also significant differences in mercury levels among the other species of fish and shellfish examined (Table 3). Large shrimp had significantly lower levels of mercury than small shrimp (χ^2^ = 7.7, *p* < 0,006), perhaps because there is growth dilution in large shrimp.

**Figure 1:**
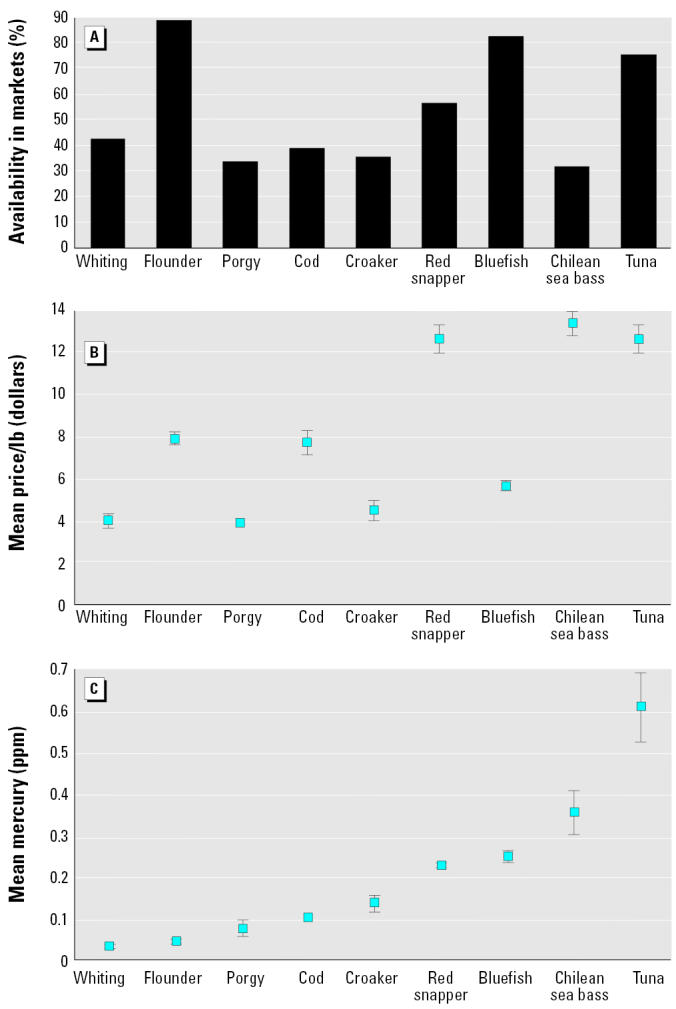
Availability (*A*), price (*B*), and total mercury levels (wet weight; *C*) in commercial fish in New Jersey (mean ± SE).

**Figure 2:**
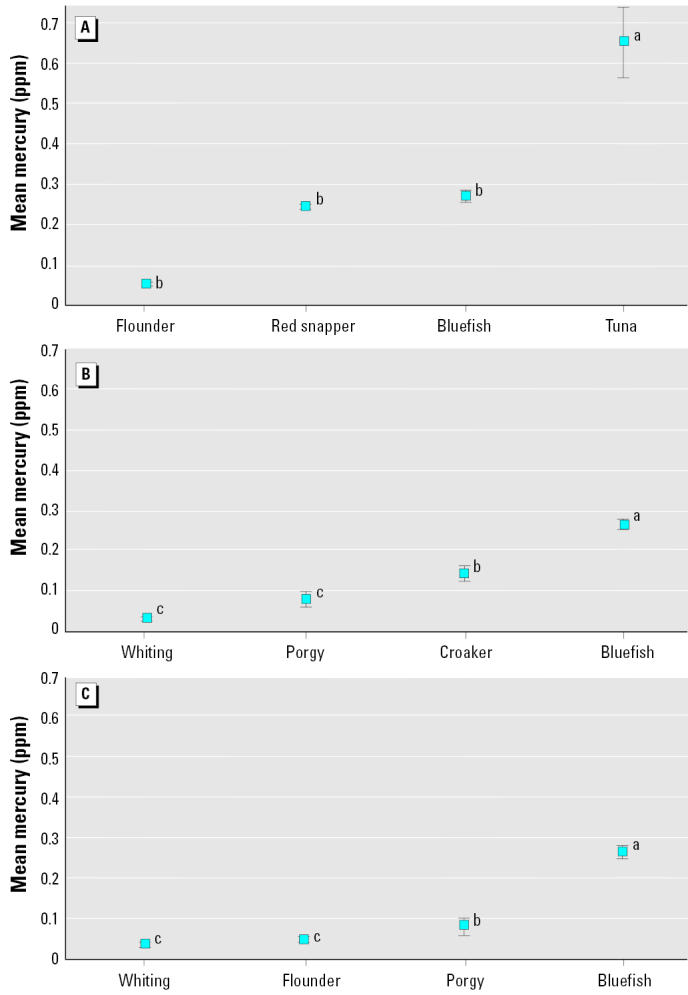
Total mercury levels (wet weight; mean ± SE) in fish if consumers selected the fish that are most available (*A*), cheapest (*B*), and optimized for price and availability (*C*). Letters that differ indicate significant differences (Duncan’s multiple range test).

Once a personal choice has been made to eat fish, the consumer must decide what types to eat. This decision may be based on several social and economic factors besides mercury concentrations, including price and availability. The fish examined in this study were not equally available in all stores, nor were they equally priced (Figure 1). Only whiting, croaker, red snapper, and tuna were available in > 50% of the stores. Fish priced < $5.00/lb ($2.27/kg) included whiting, porgy, croaker, and bluefish. If consumers selected the fish that were most available, there was a range of potential mercury exposures. If consumers selected on the basis of cost, then the range of mercury levels in these fish were even lower (Figure 2). Consumers who consistently selected the fish that were the most available and the lowest priced would select whiting, flounder, porgy, and bluefish, with bluefish having the highest mercury values (Figure 2).

## Discussion

### Mercury levels in commercial fish.

Other than the mercury levels in commercial fish and shellfish reported by the FDA (2004), there are few peer-reviewed, published articles that give mercury levels. In one article reporting mercury levels in canned tuna (Burger and Gochfeld 2004), total mercury levels averaged 0.37 ppm for white tuna and 0.118 ppm for light tuna. Since the FDA only presents means and ranges, but no measures of variation, a detailed statistical comparison is not possible. However, the comparison of means is still instructive (FDA 2004; Table 4). For most species of fish we tested, the New Jersey data showed somewhat higher mean mercury levels (even accounting for the FDA data as methyl mercury). The discrepancies could be due to year (fish for this paper were collected in 2003, compared to 1990–1992 for most FDA data), differences in the source (New Jersey may get its fish from local areas with higher levels of mercury in the marine waters), lumping data for many years, or differences in the sizes of the fish (larger fish usually have higher mercury levels) (Bidone et al. 1997; Burger et al. 2001b; Lange et al. 1994). For example, tuna can come from many different oceans, be different species of tuna, and larger individuals accumulate higher levels of mercury than smaller ones. We anticipated that mercury levels might have declined over time due to over-harvesting of large individuals and a shift to harvesting smaller individuals. The FDA database appears to be cumulative from work from 1990 to 1992, and the discrepancies suggest that the FDA and state governments should undertake a broad spectrum survey of mercury and other contaminants in fish to update their database. Further, national averages, as computed by the FDA, include the normal variation found in the regions sampled. From a state regulatory perspective, data that show discrepancies between local data and the FDA data (i.e., fresh tuna) suggests that site-specific data may be required before consumption information or advisories are prepared.

Most of the risk assessments for fish consumption examine chronic exposure, and not a single meal. However, there is recent concern that one meal of fish with a very high mercury content (a pulsed exposure) might adversely impact a developing fetus at a critical developmental period. Ginsberg and Toal (2000) have suggested that there may be risk during pregnancy for even a single-meal exposure, particularly for fish with levels of > 2.0 ppm. In the present study, we found that only tuna fillets had > 2 ppm mercury. We report the percentage of fillets that had levels > 0.5 ppm because of the need to know the percentage of times an exposure in a single meal may approach the tolerable daily intake (Berti et al. 1998). Providing information on risk from single-meal exposures, especially for pregnant women, is a public health communication challenge.

### Balancing risk with availability and price.

People are faced with making rational decisions about whether to eat fish or not and what fish to eat. Their choices are influenced by both the benefits and the risks of consuming fish (Egeland and Middaugh 1997; Knuth et al. 2003; Ponce et al. 2000) and by countervailing risks of consuming red meat compared to fish. Their choice not only depends on the available information and their own personal state (e.g., pregnant or not, thinking of becoming pregnant), but it is limited by both availability of different kinds of fish and shellfish, and at least for many Americans, price. Remarkably, although some studies have examined fish consumption as a function of seasonal availability of fish, fish quality, and education and income of the consumer (Bose and Brown 2000; Trondsen et al. 2003), studies have not examined availability and price of fish as a variable in the types of fish consumed. To our knowledge, ours is the first study that examines mercury levels in commercial fish within a context of availability and price for a geographical region the size of New Jersey.

Many of the fish and shellfish examined in this study had levels of mercury < 0.10 ppm and would pose little risk to a developing fetus. Our data suggest that consumers have choices of both shellfish and fish with low mercury levels, and such information should be provided to the public. Information on mercury levels in commercial fish will also be useful to the public in balancing the risks from self-caught and commercial fish. That is, with information on mercury (or other contaminants) in fish from their local lakes or streams, anglers or the family cook can determine whether to eat commercial or self-caught fish and how much of each species to eat. We are a long way from having sufficient information on mercury for people to make these decisions, but we suggest that agencies should go in this direction. From a public health standpoint, commercial fish is the main point of intervention to reduce methyl mercury exposure in the public.

### Risk communication.

Risk communication is effective only if the intended message reaches the audience, and if people have acquired sufficient information to feel that they are making informed decisions. Public health officials also hope that risk communication changes behavior in the desired direction. Yet people cannot make rational decisions about whether to eat fish and what kinds of fish to eat unless they have information on the risks from different choices. In our view, this means knowing not only which fish have high levels of mercury—the communication the FDA and states provide—but information on fish species that usually have low contaminant levels. Although some mercury data have been available for many years, only recently have the concentrations of omega-3 fatty acids in different fish been publicized.

It has so far proven easier for agencies to promulgate advisories that tell the public or at-risk audiences what fish not to eat than to advise them about what species of fish are low in contaminants and therefore good to eat. There are several reasons this may be true. First, contaminant analyses are expensive and time-consuming, and agencies concentrate their effort where there is a known or suspected risk. Second, advising people not to eat a fish when contaminant levels have actually declined does not have the same potential adverse effect as telling people to eat a fish that turns out to have high levels (in other words, the cost of being wrong is lower). Third, telling people that one or two species of fish are low in contaminants, while not addressing others, may pose a problem in terms of the marketplace or industry equity, and, finally, the availability of different species of fish differs among geographical regions of the state, and contaminant data on the commonly available fish will be most useful. A regional breakdown is not available in the FDA data (FDA 2004; Yess 1993).

Public health officials and appropriate state agencies should consider making available to the public information on fish that are low in mercury. This would balance the information that is currently available on fish that are high in mercury and allow people to continue to eat fish (often in large quantities) without undue harm to themselves or their children. In addition, there are ethnic preferences in fish (Burger et al. 1999, 2004), and these should be taken into account in obtaining contaminant information to disseminate to the public. Finally, the way fish are labeled is not always accurate. Many species from different parts of the world may be sold under a common rubric such as tuna or flounder. For example, a molecular analysis of fish sold as red snapper revealed that only 45% were actually that fish (Consumer Reports 2001).

We suggest that state agencies responsible for the health of their citizens conduct three kinds of studies: *a*) fish preferences of consumers as a function of economic, social, and ethnic background; *b*) fish availability in different regions and in different economic strata; and *c*) contaminant levels using a suite of fish that optimize for trophic level, consumer preferences, and market availability. This information could then be made available for the state overall, to specific geographical regions, and to different target audiences. With such information, people can make informed decisions about the species of fish to eat within their region and incomes. People’s perceptions, needs, and values with respect to fish consumption are only one part of the equation; the affected communities themselves should be involved in every step of the fish consumption advisory process (Burger 2000; Burger et al. 2003; Jardine 2003; Jardine et al. 2003). That is, stakeholders should be involved in determining which fish to analyze for mercury levels, and how risk information about specific fish should be communicated within their communities.

People do not necessarily respond similarly to positive and negative information (Liu et al. 1998), suggesting that considerable thought should go into how to present data on contaminants. Liu et al. (1998) found that people respond more quickly to negative media coverage than to positive information; but the effect of negative coverage was reduced by positive information relative to consumption. Knuth et al. (2003) showed that people would change their behavior if they were presented with risk/risk and risk/benefit information about fish consumption. In their study, the questionnaire described the health benefits and risks from consuming fish, rather than examining general knowledge. Appropriate changes in behavior are possible only if people have knowledge of the nature of the risks for a range of species, allowing them to choose what they wish to eat. We also suggest that similar information be available on the benefits of specific fish, including levels of omega-3 fatty acids.

## Conclusions

Overall, we found no significant differences in mercury levels in tuna, bluefish, and flounder as a function of type of store or economic neighborhood, except that flounder from fish markets along the Jersey shore had higher levels of mercury than flounder bought in other markets. Flounder from shore markets came from very local sources, whereas for the other regions the source of fish may have been from regional fish markets or distribution centers. There were significant differences in mean mercury levels in the fish and shellfish examined. Further, for tuna, sea bass, croaker, whiting, and shrimp, the levels of mercury were higher in New Jersey samples than those reported by the FDA (2004). This suggests that regional differences in mercury levels should be reported when national data on mercury levels are aggregated, allowing state agencies to evaluate possible risk for their citizens. It may also be useful to obtain information on levels of mercury as a function of the source of commercial fish, as well as seasonal trends.

There were significant differences in availability (and cost). We found that consumers optimizing for easy availability would select flounder, snapper, bluefish and tuna, whereas those selecting only for price would select whiting, porgy, croaker, and bluefish. Flounder demonstrated the best relationship among availability, cost, and low mercury levels. We suggest that agencies responsible for protecting human health should obtain information on fish availability and cost in markets, as well as fish preferences, and use this information to select fish for analysis of contaminant levels. This would provide data on the most commonly eaten fish. Public health officials could then provide the public with information on mercury, cost, and availability for commercial fish, allowing them to make informed decisions about which fish to eat.

## Correction

In the original manuscript published online, the authors stated that they found “single fillets of tuna, Chilean sea bass, croaker, and red snapper that had > 2 ppm mercury.” This statement has been corrected here to indicate that “only tuna fillets had > 2 ppm mercury.”

## Figures and Tables

**Table 1:** Mercury levels (ppm, wet weight) in commercial fish from New Jersey markets sampled in 2003.

	Tuna	Bluefish	Flounder
Overall sample size (*n*)	50	53	55
Overall means	0.6 ± 0.1	0.3 ± 0.02	0.05 ± 0.01
New Jersey region			
North	0.8 ± 0.2 (12)	0.2 ± 0.02 (15)	0.05 ± 0.01 (17)
Central	0.8 ± 0.2 (16)	0.3 ± 0.02 (16)	0.03 ± 0.01 (16)
South	0.5 ± 0.1 (16)	0.3 ± 0.03 (16)	0.05 ± 0.01 (16)
Shore	0.4 ± 0.1 (6)	0.4 ± 0.09 (6)	0.07 ± 0.02 (6)
χ^2^ (*p*)	NS	NS	8.8 (0.03)
Type			
Supermarket	0.8 ± 0.2 (21)	0.2 ± 0.02 (20)	0.05 ± 0.01 (21)
Market	0.5 ± 0.1 (29)	0.3 ± 0.02 (33)	0.05 ± 0.01 (34)
χ^2^ (*p*)	2.8 (0.09)	NS	NS
Socioeconomic status			
High	0.6 ± 0.1 (29)	0.3 ± 0.02 (27)	0.05 ± 0.007 (26)
Low	0.7 ± 0.1 (21)	0.2 ± 0.02 (26)	0.04 ± 0.006 (26)
χ^2^ (*p*)	NS	NS	NS

NS, not significant. Values shown are mean ± SE (*n*) except where shown.

**Table 2:** Overall levels (ppm, wet weight) of mercury in fish collected throughout New Jersey.

	Tuna	Bluefish	Flounder
Sample size (*n*)	50	53	55
Mean ± SE	0.6 ± 0.1	0.3 ± 0.02	0.05 ± 0.01
Geometric mean	0.4	0.2	0.04
Low value	0.084	0.009	0.002
High value	2.5	0.76	0.14
Percent > 0.3 ppm	62	32	0
Percent > 0.5 ppm	42	2	0
Percent > 0.75 ppm	26	2	0

**Table 3:** Mercury levels (ppm, wet weight) in commercial fish from New Jersey markets (sampled in 2003).

Species (*n*)	Mean ± SE	Geometric mean	Minimum	Maximum
Chilean sea bass (7)	0.4 ± 0.1^a^	0.3	0.2	0.6
Red snapper (4)	0.2 ± 0.01^b^	0.2	0.2	0.3
Cod (7)	0.1 ± 0.006^c^	0.1	0.08	0.1
Croaker (14)	0.1 ± 0.02^c^	0.1	0.06	0.3
Porgy (14)	0.08 ± 0.02^c^	0.08	0.02	0.2
Whiting (14)	0.03 ± 0.004^d^	0.03	0.006	0.1
Scallops (12)	0.01 ± 0.001^d^	0.012	0.007	0.02
Shrimp, small (12)	0.02 ± 0.001^d^	0.01	0.008	0.02
Shrimp, large (12)	0.01 ± 0.001^d^	0.01	0.002	0.02
χ^2^ (*p*)	81 (0.0001)			

Different letters indicate significant differences by Duncan’s multiple range test; the same letter indicates no difference between means.

**Table 4:** Comparison of mercury concentrations (ppm) in fish from the FDA (2004) and from the present study.

Species	Present study [mean ± SE (*n*)]	FDA (2004) [mean (*n*)]
Tuna (fresh)	0.64 ± 0.09 (50)	0.38 (131)
Chilean sea bass	0.38 ± 0.02 (7)	0.27 (35)
Bluefish	0.26 ± 0.02 (53)	0.31 (22)
Porgy	0.08 ± 0.02 (14)	—*^a^*
Red snapper	0.24 ± 0.01 (4)*^b^*	0.19 (25)
Croaker	0.14 ± 0.02 (14)	0.05 (21)
Cod	0.11 ± 0.06 (7)	0.11 (20)
Flounder	0.05 ± 0.01 (55)	0.05 (22)
Whiting	0.03 ± 0.04 (14)	ND (2)
Scallop	0.01 ± 0.00 (12)	0.05 (66)
Shrimp	0.02 ± 0.00 (24)	ND (24)
Tuna (canned albacore)	0.37 ± 0.02 (123)*^c^*	0.35 (179)

ND, not detectable. Our values are total mercury, but FDA (2004) values are sometimes given as total mercury and sometimes as methyl mercury. A subset analyzed for methyl mercury indicated that methyl mercury is 89% of total mercury, at least for canned tuna (Burger and Gochfeld 2004).

a Not examined.

b In a 2000 sample of 80 fish, we obtained lower values for mercury.

c Results from Burger and Gochfeld (2004) from our laboratory.
